# Supplementary figures and images for: Gene duplicates cause hybrid lethality between sympatric species of Mimulus
Source: PLoS Genet. 2018 Apr 12;14(4):e1007130. doi: 10.1371/journal.pgen.1007130 (PMC5896889; doi:10.1371/journal.pgen.1007130)

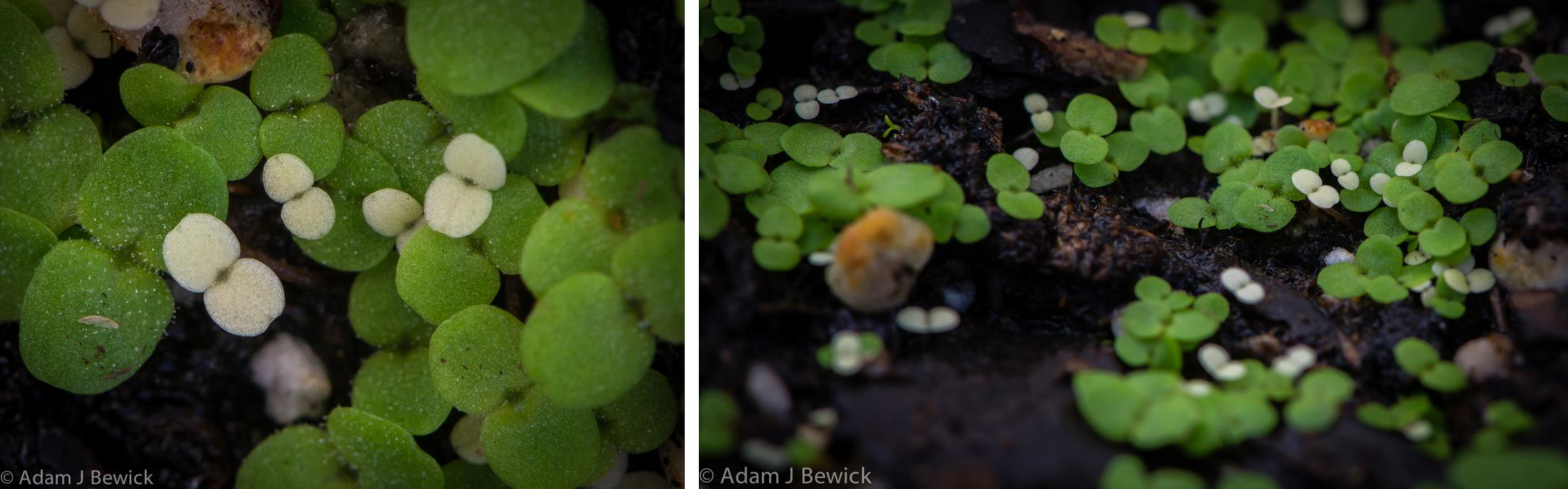

Supplement: S1 Fig — White seedlings segregate in 1:15 in reciprocal F2 hybrids of M. guttatus (DPR102-gutt) and M. nasutus (DPR104-nas). Photos kindly provided by Adam J Bewick. (TIF) [file pgen.1007130.s001.tif]

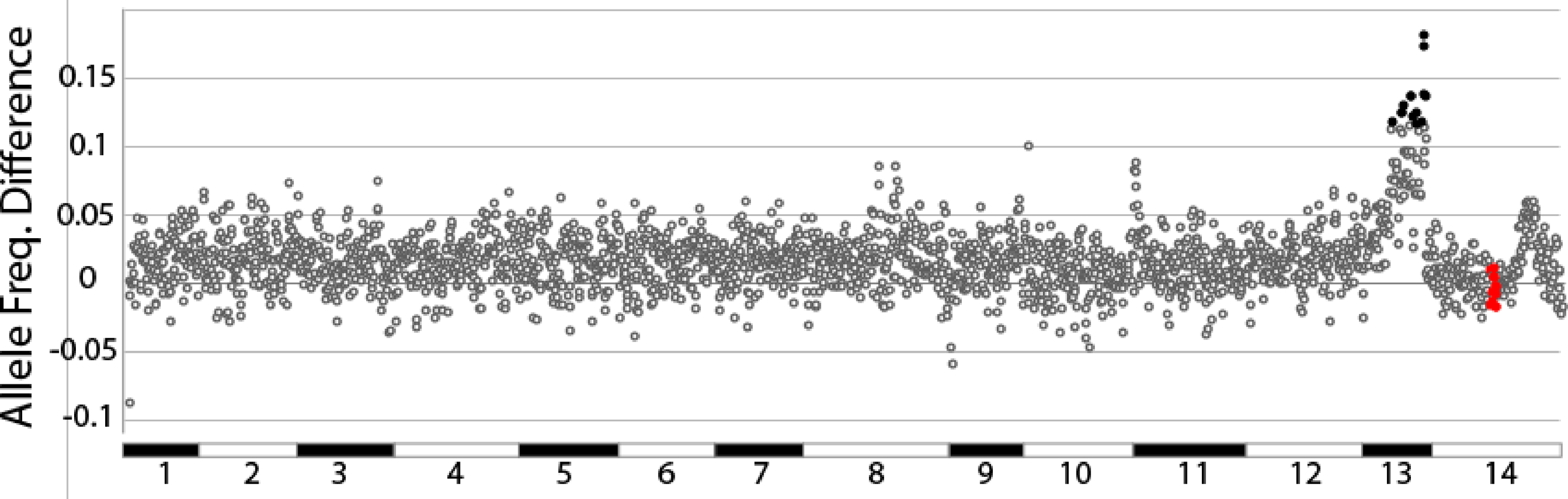

Supplement: S2 Fig — Difference in average allele frequency between green and white pools (plotted along the fourteen Mimulus chromosomes) was calculated in 200-SNP windows with 100-SNP overlap. The 0.5% most divergent windows are highlighted as black dots, which are all located at the distal end of chromosome 13 in contiguous windows and represent the candidate hl13 region. Red dots, which overlap with the previously mapped interval for hl14, show the 0.5% least divergent windows (calculated as absolute difference in allele frequency). (TIF) [file pgen.1007130.s002.tif]

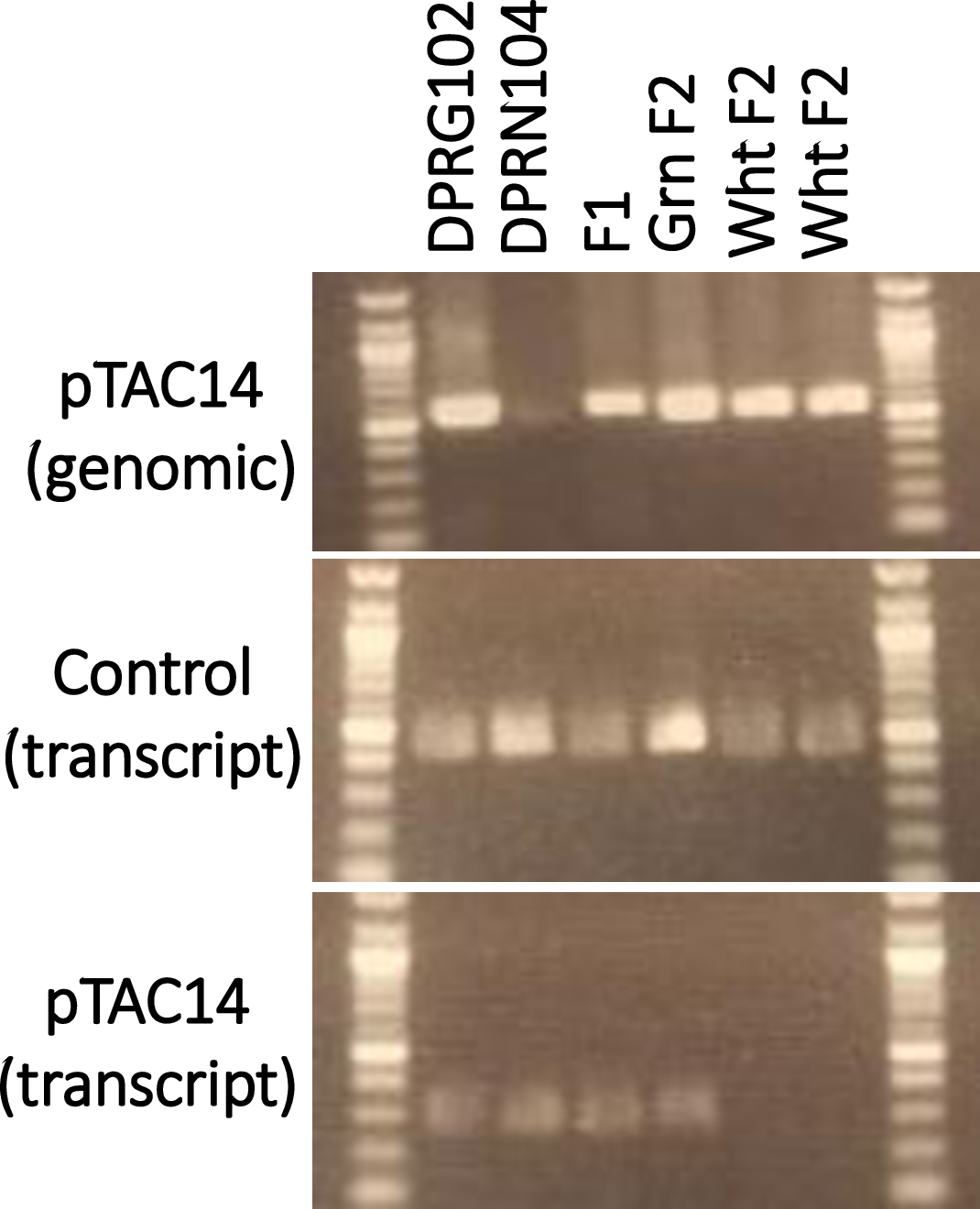

Supplement: S4 Fig — PCR products run on 1% agarose gel with 2-log ladder. Control transcript is Migut.M00195 (ACYL-COENZYME A OXIDASE-LIKE PROTEIN). Note that DNA and RNA was extracted from pools of 10 seedlings for each genotype. (TIF) [file pgen.1007130.s004.tif]

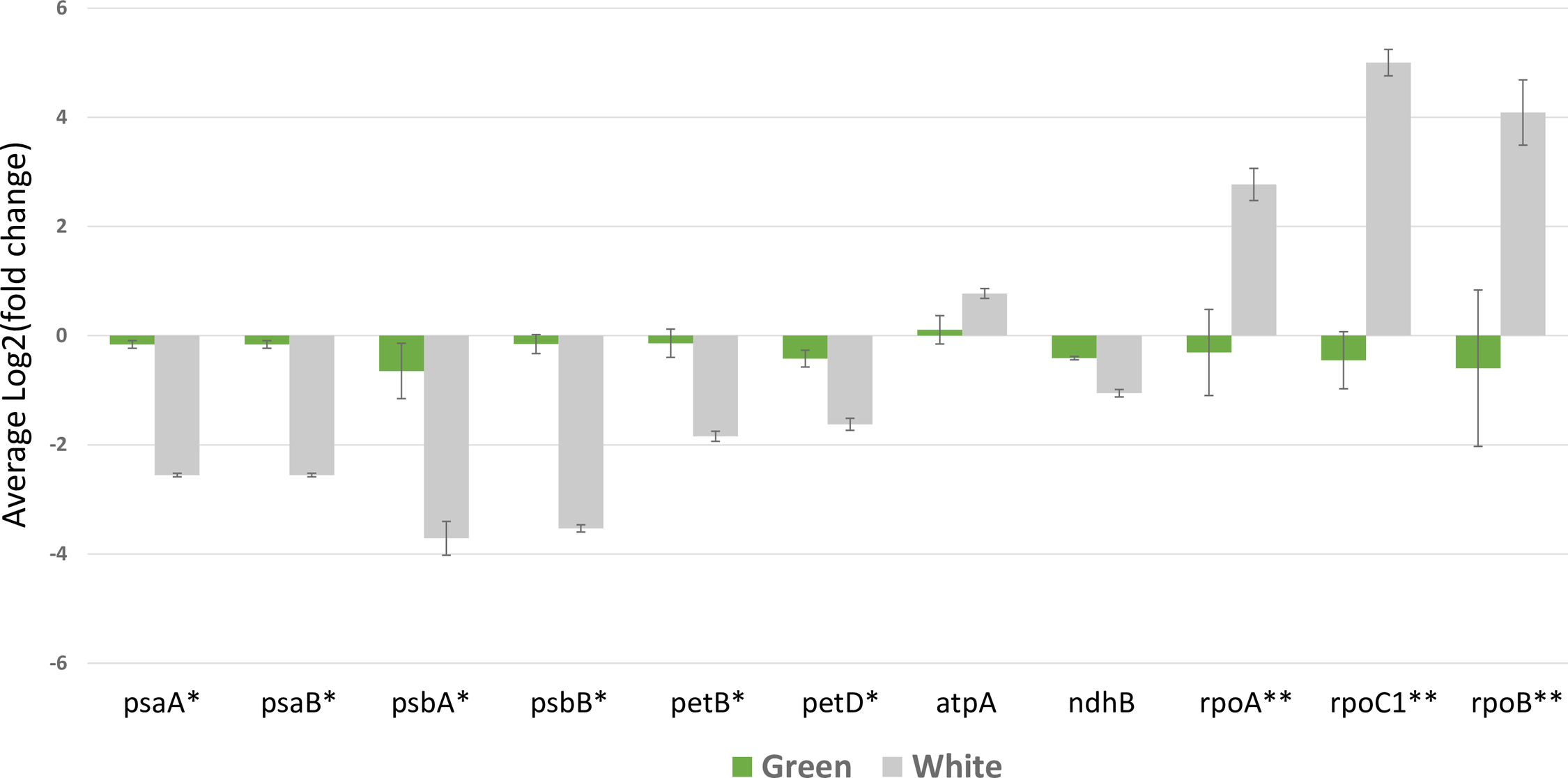

Supplement: S5 Fig — Differential expression among chloroplast genes transcribed by PEP (psaA, psaB, psbA, psbB, petB, petD), PEP and NEP (atpA and ndhB), and NEP (rpoA, rpoB, and rpoC1). Green: average log2 fold-change in all pairwise comparisons among green seedlings (DPR102-gutt, DPR104-nas, Green F2). White: average log2 fold-change in pairwise comparisons between white F2 seedlings and green seedlings. *Significantly down-regulated in white seedlings (all pairwise comparisons, p<5.0−5). **Significantly up-regulated in white seedlings (all pairwise comparisons, p<5.0−5). (TIF) [file pgen.1007130.s005.tif]

**Table S3:** GO term enrichment of differentially expressed genes.


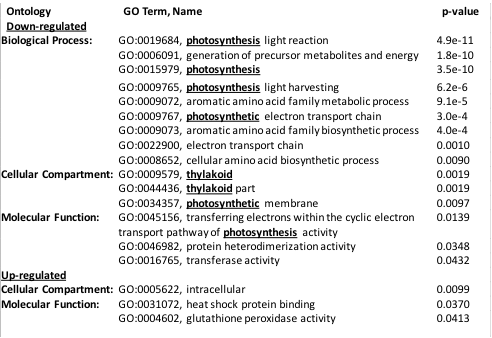

Supplement: S3 Table — (DOCX) [file pgen.1007130.s008.docx]
