# Supplementary material for: Gene duplicates cause hybrid lethality between sympatric species of Mimulus
Source: PLoS Genet. 2018 Apr 12;14(4):e1007130. doi: 10.1371/journal.pgen.1007130 (PMC5896889; doi:10.1371/journal.pgen.1007130)
Supplement: S1 Table — (DOCX) [file pgen.1007130.s006.docx]

**Table S1:** **Segregation of white seedlings in parental and reciprocal hybrid crosses.** Maternal parents are listed first in crosses. ‘G’ is DPRG102 (*M. guttatus*) and ‘N’ is DPRN104 (*M. nasutus*). *Χ*^2^ tests with one degree of freedom used to assess green:white ratios expected under various genetic models: 2 recessive alleles (15:1), dominant-recessive (13:3), 3 recessive alleles (63:1), and 2 recessive and 1 dominant (61:3). Non-significant values (bold) indicate models consistent with observed ratios.

| Cross | # Green | # White | 2-loci (15:1) | 2-loci (13:3) | 3-loci (63:1) | 3-loci (61:3) |
| --- | --- | --- | --- | --- | --- | --- |
| GxG | 534 | 0 | 35.6*** | 123.23*** | 8.46** | 26.26*** |
| NxN | 355 | 0 | 23.66*** | 81.92*** | 5.63* | 17.46*** |
| GxN F1 | 634 | 0 | 42.26*** | 146.31*** | 10.06** | 31.18*** |
| NxG F1 | 577 | 0 | 38.47*** | 133.15*** | 9.16** | 28.38*** |
| GxN F2 | 478 | 38 | **1.09** | 43.91*** | 112.92*** | 8.28** |
| NxG F2 | 623 | 38 | **0.28** | 73.34*** | 75.32*** | **1.67** |

*=P<0.05; **=P<0.01; ***=P<0.001
