# Supplementary material for: Gene duplicates cause hybrid lethality between sympatric species of Mimulus
Source: PLoS Genet. 2018 Apr 12;14(4):e1007130. doi: 10.1371/journal.pgen.1007130 (PMC5896889; doi:10.1371/journal.pgen.1007130)
Supplement: S2 Table — (DOCX) [file pgen.1007130.s007.docx]

**Supplementary Table 2:** Candidate genes at *hl13* and *hl14*

| **Gene** | **Annotation** | **Predicted Function** | **Chlorosis/lethality phenotype in *Arabidopsis*** |
| --- | --- | --- | --- |
| ***hl13*** |  |  |  |
| Migut.M02005 | Allantoate deiminase | Purine turnover | None |
| Migut.M02006 | None | ATP binding, carbohydrate binding, kinase activity | None |
| Migut.M02007* | Purine biosynthesis protein 6/7, PUR6/7 | Catalyzes aspartate addition to alpha-amino group to purine backbone | Localized to chloroplast, chloroplast stroma |
| Migut.M02008 | Zinc finger protein 207 | Encodes SUF4, a putative transcription factor required for delayed flowering in winter-annual | None |
| Migut.M02009 | Protein unusual floral organs | Required for proper identity of floral meristem. | None |
| Migut.M02010 | GTP-binding protein ERG | RNA binding, GTP binding | None |
| Migut.M02011 | DNA polymerase ETA | Catalyses translesion synthesis in response to UV damage. | None |
| Migut.M02012 | Phosphoglycerate mutase family protein | Catalytic activity | None |
| Migut.M02013 | Proteosome subunit alpha type-4 | Encodes the alpha-3 subunit of 20S proteasome | None |
| Migut.M02014 | Haloacid dehalogenase-like hydrolase | Hydrolase, catalytic, and phosphoglycolate phosphatase activity | None |
| Migut.M02015 | Mitochondrial translocator assembly and maintenance protein 41 | None | Localized to chloroplast |
| Migut.M02016 & Migut.M02019 | Cationic amino acid transporter 5 | Amino acid transporter | None |
| Migut.M02017 & Migut.M02020 & Migut.M02022 | 40S ribosomal protein S28 | Structural constituent of ribosome, mRNA binding | None |
| Migut.M02018* | Mitochondrial amidoxime-reducing component 1 | Molybdenum ion binding, catalytic activity | Localized to chloroplast |
| Migut.M02021 | Myb-like DNA binding domain | Telomeric repeat binding protein | None |
| Migut.M02023* | N-lysine methyltransferase EFM1; pTAC14 | Regulatory role in chloroplast development and gene expression | Localized to chloroplast. Chloroplast fails to develop in knockout mutants, leading to premature lethality at the cotyledon stage. |
| Migut.M02024 | Vacuolar-sorting receptor 6 | Calcium ion binding, protein targeting to vacuole | None |
| Migut.M02025* | Protein trigalactosyldiacylglycerol 2, chloroplastic | Involved in lipid transfer from ER to chloroplast. | Mutants consistently smaller and slightly pale, with reduced chlorophyll content. |
| Migut.M02026* | PPR repeat family | Involved in chloroplast mRNA editing. | Localized to chloroplast. |
| Migut.M02027 | Myb-like DNA-binding protein | None | None |
| Migut.M02028 | Transducin/WD40 domain-containing protein | None | None |
| Migut.M02029 | LRR receptor-like serine/threonine protein kinase GS01-related | Required for formation of normal epidermal surface during embryogenesis | None |
| ***hl14*** |  |  |  |
| Migut.N01485 | Clustered mitochondria protein | Regulates mitochondrial association time and involved in mitochondrial fusion | Numerous phenotypes including lower biomass, shorter roots, short etiolated hypocotyls, large number of dead root cells, and overall transcriptome reprogramming. |
| Migut.N01486 | DNAJ homolog subfamily C member | Involved in female gametophyte development | None |
| Migut.N01487* | C2 calcium/lipid binding plant phosphoribosyltransferase family protein | Involved in transport of florigen FT from companion cells to sieve elements | None |
| Migut.N01488* | Sister chromatid cohesion 1 protein 3 | Essential for megagametogenesis and plays important role in pollen development, a key component of meiotic and mitotic cohesion complexes | Gametophytic lethality phenotypes in male and female gameotphytes – no evidence for seed development defects |
| Migut.N01489 | Thioredoxin superfamily protein | Oxidation-reduction process | None |
| Migut.N01490 | AXI 1 protein-like protein | Transferase activity | None |

*Genes that were analyzed in our qPCR experiment.
